# Evaluation of parameters affecting gamma passing rate in patient‐specific QAs for multiple brain lesions IMRS treatments using ray‐station treatment planning system

**DOI:** 10.1002/acm2.13467

**Published:** 2021-11-18

**Authors:** Elahheh Salari, E. Ishmael Parsai, Diana Shvydka, Nicholas Niven Sperling

**Affiliations:** ^1^ Department of Radiation Oncology University of Toledo Medical Center Toledo Ohio USA

**Keywords:** energy, IMRS, planned dose grid, VMAT

## Abstract

**Purpose:**

Using intensity‐modulated radiosurgery (IMRS) with single isocenter for the treatment of multiple brain lesions has gained acceptance in recent years. One of the challenges of this technique is conducting a patient‐specific quality assurance (QA), involving accurate gamma passing rate (GPR) calculations for small and wide spread‐out targets. We evaluated effects of parameters such as dose grid and energy on GPR using our clinical IMRS plans.

**Methods:**

Ten patients with total of 40 volumetric modulated arc therapy (VMAT) plans were created in Raystation (V.8A) treatment planning system (TPS) for the Varian Edge Linac using 6 and 10 flattening filter‐free (FFF) beams and planned dose grids of 1 mm and 2 mm resulting in four plans with 6–10 targets per patient. All parameters and objectives except dose grid and energy were kept the same in all plans. Next, patient‐specific QAs were measured evaluating GPR with 10% threshold, 3%/3 mm objective, and an acceptance criterion of 95%. Modulation factors (MF) and confidence intervals were calculated. Two modes of measurements, standard density (SD) and high density (HD), were used.

**Results:**

Generally, plans computed with 1 mm dose grid have higher GPRs than those with 2 mm dose grid for both energies used. The GPRs of 6 FFF plans were higher than those of 10 FFF plans. GPR showed no noticeable difference between HD and SD measurements. Negative correlation between MF and GPR was observed. The HD pass rates fall within the confidence interval of SD.

**Conclusion:**

Calculated dose grid should be less than or equal to one‐third of distance to agreement, thus 1 mm planned dose grid is recommended to reduce artifacts in gamma calculation. GPR of SD and HD measurement modes is almost the same, which indicates that SD mode is clinically preferable for performing patient‐specific QAs. According to our results, using 6 FFF beams with 1 mm planned dose grid is more accurate and reliable for dose calculation of IMRS plans.

## 1 INTRODUCTION

Intracranial metastatic disease is one the most frequent neurological complications of primary cancer.^1^ Different methods have been used to treat multiple intracranial metastases such as whole brain radiation therapy (WBRT), stereotactic radiosurgery (SRS), Gamma Knife radiosurgery, and recently single isocenter multiple target (SIMT) SRS. Due to rapid development of real‐time image‐guided radiotherapy (IGRT) and cone beam CT (CBCT) for localizing targets, the treatment of multiple metastases using a C‐arm Linac has gained acceptance. Planning can be accomplished using a single‐isocenter technique combined with volumetric modulated arc therapy (VMAT) optimization to generate clinically suitable and deliverable intensity‐modulated radiosurgery (IMRS) treatment plans.^2^, ^3^

This technique, however, has some challenges and limitations that require more investigation. One of the significant challenges is pretreatment quality assurance (QA) verification, required for inverse‐planned treatments. By design IMRS involves multiple beams delivering highly modulated planned radiation dose to small and wide spread‐out targets. The treatment isocenter is placed in a location central to all targets and not inside of any specific target, further complicating the measurement of dose distribution for individual beams.

A common tool for comparison between the calculated and the measured dose for a planar dose distribution is the gamma passing rate (GPR), calculated from the gamma values described by Low et al. ^4^ The purpose of this work is to evaluate effects of different parameters such as grid size (GS), detector resolution, and treatment energy on GPR of our IMRS plans.

## 2 MATERIALS AND METHODS

**FIGURE 1:**
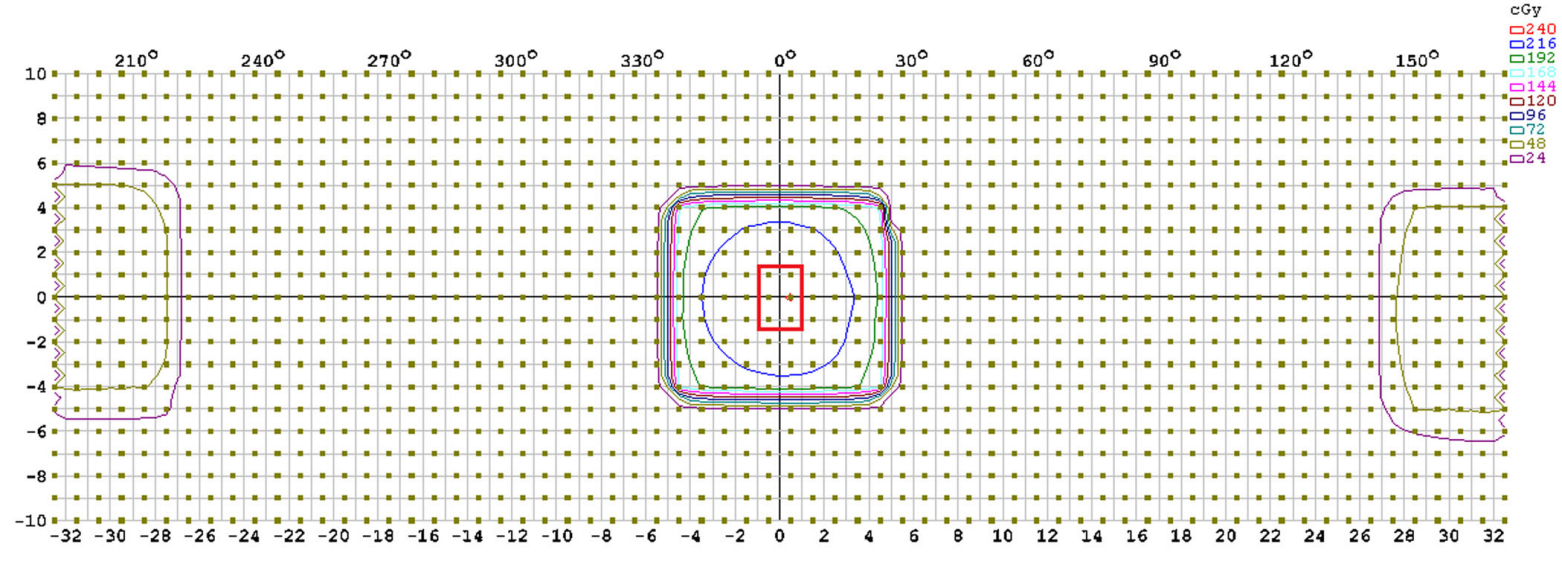
Six diodes in the red rectangular area within 5 mm of the central axis of ArcCHECK phantom used for dose rate dependence, angular dependence, and Rotisserie test

A pretreatment VMAT plan QA verification typically involves use of an appropriate pre‐treatment QA dosimetry phantom, capable of acquiring both the absolute dose and spatial dose distribution. In our clinic, ArcCHECK phantom and its associated software (SNC Patient) were utilized. This software allows correcting for angular dependence, inhomogeneity, and field size dependence, as well as merging measurements to create high‐density (HD) dose distribution from multiple individual standard‐density (SD) measurements. The delivery of radiation using the VMAT technique involves the accelerator gantry rotating around the patient while dose rate, gantry speed, and MLC positions are changing dynamically. In order to characterize the ArcCHECK phantom output for these conditions, the dose rate, angular dependency, and symmetry response of the ArcCHECK must be evaluated prior to performing any patient‐specific QA. We closely examined the average measured doses of the six diodes nearest to the center of the irradiated field on the ArcCHECK phantom for this analysis (Figure 1). To minimize effects of gantry speed deviation, evaluation was performed at static gantry angles of 0°, 30°, 60°, 90°, 120°, 270°, 300°, and 330°.

### 2.1 Dose‐rate and angular dependence of ArcCHECK phantom

The dose rate and angular dependence of the ArcCHECK were evaluated for the dose rates of 400, 600, 800, 1000, and 1200 MU/min for 6 MV flattening filter‐free (FFF) and 400, 800, 1200, 1600, 2000, and 2400 MU/min for 10 MV FFF delivering 200 MU to a 10 × 10 cm^2^ static field. The phantom was set up in the typical measurement conditions at 100 cm SAD to the phantom center.

### 2.2 Rotisserie test (symmetry of response with phantom rotated axially)

For accurate evaluation, the interdiode variability on the ArcCHECK phantom, which has a symmetrical detector arrangement in a helical pattern, should be corrected by the software to give the same results for any beam angle exposure. In a previous study by Feygelman et al., they described a test to evaluate the phantom symmetry response with respect to axial rotation.^5^ To evaluate our phantom, an open field 10 × 10 cm^2^, 6 MV FFF beam was delivered to 200 MU at 1400 MU/min with the gantry angle fixed at 0°. The phantom was irradiated seven times with 30° phantom rotation between the irradiations. The dose delivered to the six diodes closest to the center of the exposed field was used to evaluate the delivered dose.

### 2.3 VMAT plans

**FIGURE 2:**
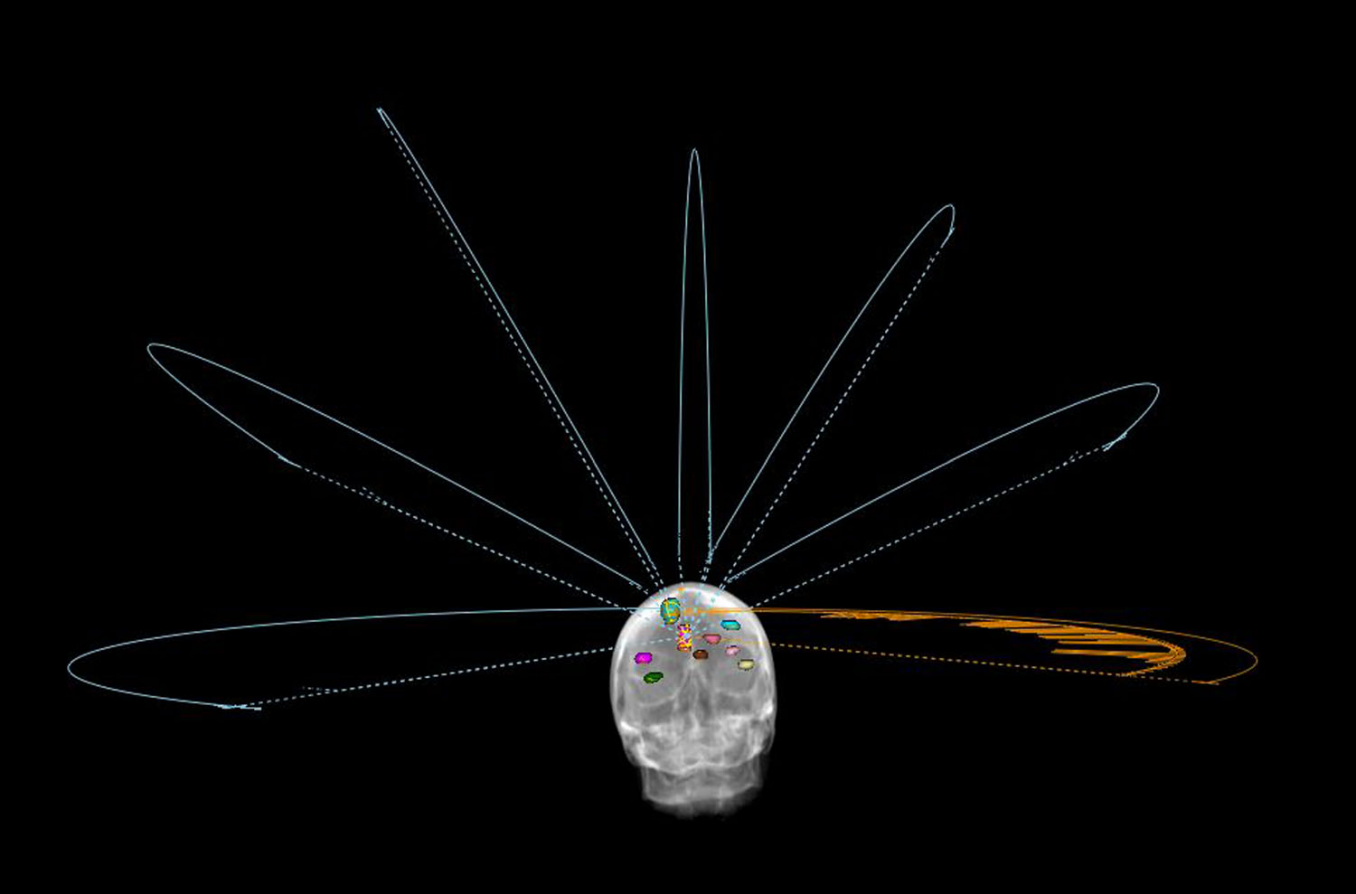
Illustration of arc beams arrangement in VMAT plans

In 2016, Morrison et al. showed dosimetry benefits of using one isocenter with 4– 6 arcs for treating multiple intracranial metastases to improve conformity index and decrease normal brain volumes encompassed by 6 Gy and 12 Gy dose levels, *V*_6Gy_, and *V*_12Gy_ correspondingly, when targets were placed within 5 cm or even more than 5 cm apart from the isocenter.^6^ In this study, 10 previously treated patient scans with 6–10 targets with averaged size of 1.2 cm^3^ (range 0.57–2.68 cm^3^) within 5 cm distance from isocenter each were used to develop SIMT treatment plans in Raystation 8A SP1 (8.0.1.10) treatment planning system (TPS) for the Varian Edge Linac. A standardized beam configuration consisting of 7 arcs of <180°, two of which are at 0° couch rotation, with the remaining 5 as equally distributed supracephalic non‐coplanar arcs (Figure 2). In our clinical experience, 10 MV FFF beam may provide more optimized dose distribution, especially to treat single isocenter multiple cranial metastases. Also, some previous studies^2^, ^7^ indicate advantages of using 10 MV FFF for this technique. Therefore, in this study, plans were generated using FFF beams, both 6 MV FFF and 10 MV FFF, with GSs of 1 mm and 2 mm resulting in four plans for each patient, amounting to 40 analyzed plans. Each plan was designed with a total prescribed dose of 24 Gy (8 Gy per fraction) to composite planning target volumes (PTV). All planning parameters and optimization objectives were kept the same across all plans. For purposes of comparison, the 6 MV FFF plan with 1 mm GS was selected as a reference plan for each patient.

### 2.4 QA of VMAT plan

QA plans were generated for all 40 treatment plans by calculating each plan on a CT scan of the ArcCHECK phantom in Raystation and recalculating doses for the phantom geometry. Then, QA files exported as DICOM files for performing patient‐specific QA. Measurements of patient‐specific QA were performed using a Sun Nuclear ArcCHECK phantom that contains 1386 Sunpoint diode detectors—each with an active element of 0.8 × 0.8 mm^2^ surface area arranged in a three‐dimensional spiral pattern with 1 cm detector spacing and 1 cm stripe width. The diameter of center of the phantom is 15 cm. The diode detector elements are sandwiched between 2.9 cm thick rings of PMMA (water equivalent thickness of 3.3 cm) buildup and backscatter. The length of the active area (detector array) is 21 cm.^8^ The setup of ArcCHECK phantom and beam delivery were based on the true composite (TC) method of TG‐218, modified to include field‐by‐field analysis instead of the composite analysis. At the end, GPRs of individual fields were averaged for each plan.

Treatment plans were delivered using a Varian Edge Linac and measured using the SNC ArcCHECK and patient software program (version 8.2). Global normalization for calculation of percent difference was used (Van Dyk criterion^9^), with the maximum point dose in each field for the planned dose used for normalization. Using global normalization of the data reduces the number of failing low dose points, which are often found outside of the treatment fields and may have small absolute dose differences causing large uncertainties.^10^, ^11^, ^12^ Following this approach, we were able to focus on revealing clinically relevant errors in patient plans. The criteria of absolute gamma analysis with 3 mm/3%, GPR of 95% and threshold of 10% were applied in QA analysis.

The SNC patient software includes a tool named “Calc shift,” which adjusts the spatial offset between planned dose and measured dose in order to minimize number of failed points.^13^ To minimize any potential setup irregularities, “Calc shift” was applied to all measured arcs individually.

### 2.5 SD and HD measurement

To address the concern regarding the relatively large detector separation and small sensing elements of the ArcCHECK phantom (detector density) undersampling the measured doses, the SNC patient software may be used to combine multiple measurements within a rotated and translated detector reference frame to create synthetically higher detector densities for measurement evaluation. As part of this study where small and widely spread‐out lesions are involved, two different modes of measurements, namely, standard density (SD) with 1386 total number of detector and high density (HD), were used. The HD measurement is a combination of a typically obtained SD measurement with another measurement obtained by translating the ArcCHECK phantom ±5 mm in axial direction and rotating ±2.72° axially. This combination results in a 0.5 cm spacing between diode detectors. All measurements were compared with TPS calculated dose distributions.

### 2.6 Second monitor unit check and modulation factor

#### 2.6.1 Second MU check

Every plan received an independent monitor unit verification calculation (MUVC) check. This is another patient‐specific dosimetry QA procedure for IMRT treatment plans suggested by TG‐114 to be done for each individual treatment field.^14^ In this study, we used a clinically commissioned machine model in RadCalc™ (LifeLine Software, Inc., LAP Group, V6.4) as an independent MUVC for checking dose accuracy calculation of all 40 VMAT plans.

#### 2.6.2 Modulation factor

Modulation factor (MF) is a dosimetric parameter used to quantify plan delivery complexity. For IMRT/VMAT plans, MU increases due to the MLC motions, gantry rotations, number of control points, and dose calculations of small or irregular fields. In this study, MF was calculated based on Equation (1) with Pearson's correlation analysis used to analyze relationship between GPR and MF:

(1) $$\mathrm{MF}\left(\frac{\mathrm{MU}}{\mathrm{cGY}}\right)\;=\;\frac{\text{Total}\;\text{MU}\;\text{per}\;\text{Plan}}{\text{Total}\;\text{Dose}\;\text{per}\;\text{Plan}\;}.$$

### 2.7 Confidence interval

The ArcCHECK phantom used in this study is considered a low‐density detector (i.e., 1 detector/cm). For this type of detector, Bailey et al.^12^ have shown that GPR is not absolute but represents statistical variability as a function of detector sampling geometry and position. To provide a measure of the uncertainty, a statistical model as proposed by Bailey et al. was used to calculate a confidence interval (CI) for SD measurements:

(2) $$\mathrm{CI}\;=\;p\pm z_{1-\frac{\alpha }{2}}\sqrt{\frac{p\left(1-p\right)}{n}\;}\sqrt{\frac{N-n}{N-1}}.$$

Here, *N* is the number of data points used for data analysis with HD mode data acquisition and *n* is the number of sampled points for low‐density SD measurement, *p* is the probability of achieving a pass, and *Z*_1‐α/2_ is 1.96 for a 95% Confidence Interval (CI).

### 2.8 Confidence limit and detectability threshold

There are always differences between measurements and calculations. These differences may happen due to limited spatial resolution of a QA device, limitation in accuracy of dose calculation, and limitation in dose delivery mechanism. Therefore, a degree of agreement between measured and calculated dose should be quantified using the concept of confidence limit (CL) as proposed by TG‐119.^15^ CL for the gamma analysis was computed based on the following formula from TG‐119:

(3) $$\mathrm{CL}\;=\left(100-\mathrm{mean}\right)\;+1.96\;\sigma ,$$

where mean is the average percentage of GPR and σ is the standard deviation.

The detectability threshold (DT) of ArcCHECK phantom for 6 MV FFF and 10 MV FFF and both GS 1 mm and 2 mm was calculated from the CL as follows:

(4) DT=100−CL.

As per TG‐119 for diode arrays, the computed statistical results obtained are stable when DT is greater than 93%.

## 3 RESULTS

### 3.1 Dose rate and angular dependence of ArcCHECK phantom

#### 3.1.1 Dose rate dependence

**FIGURE 3:**
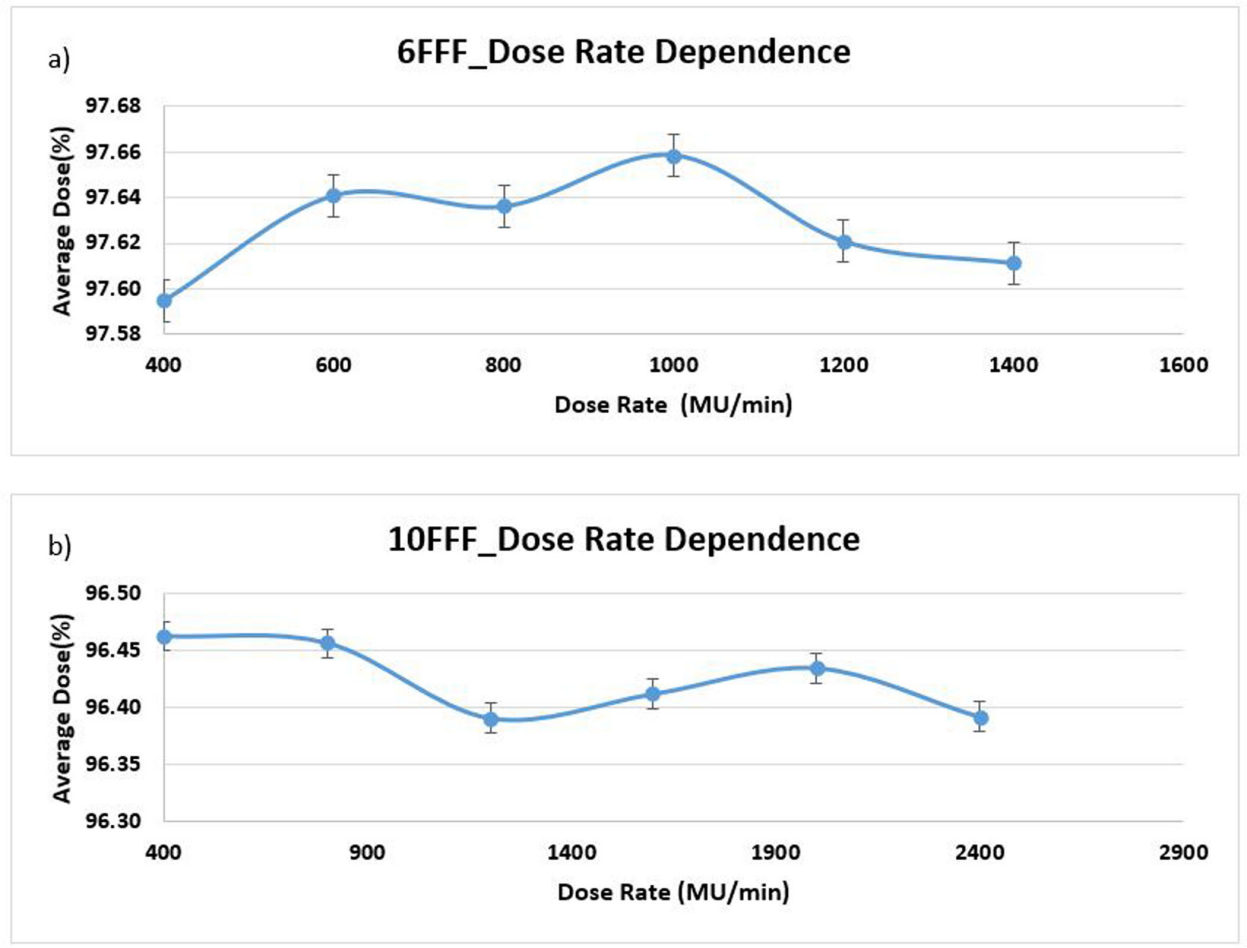
Dose rate dependence at gantry angle 0: (a) 6 MV FFF and (b) 10 MV FFF with maximum deviation of 0.063 and 0.061 for 6 MV FFF and 10 MV FFF, respectively

Dose rate dependency was measured at a gantry angle of 0°. Maximum deviation was 0.063 for 6 MV FFF and 0.061 for 10 MV FFF, as shown in Figure 3.

#### 3.1.2 Angular dependence

**FIGURE 4:**
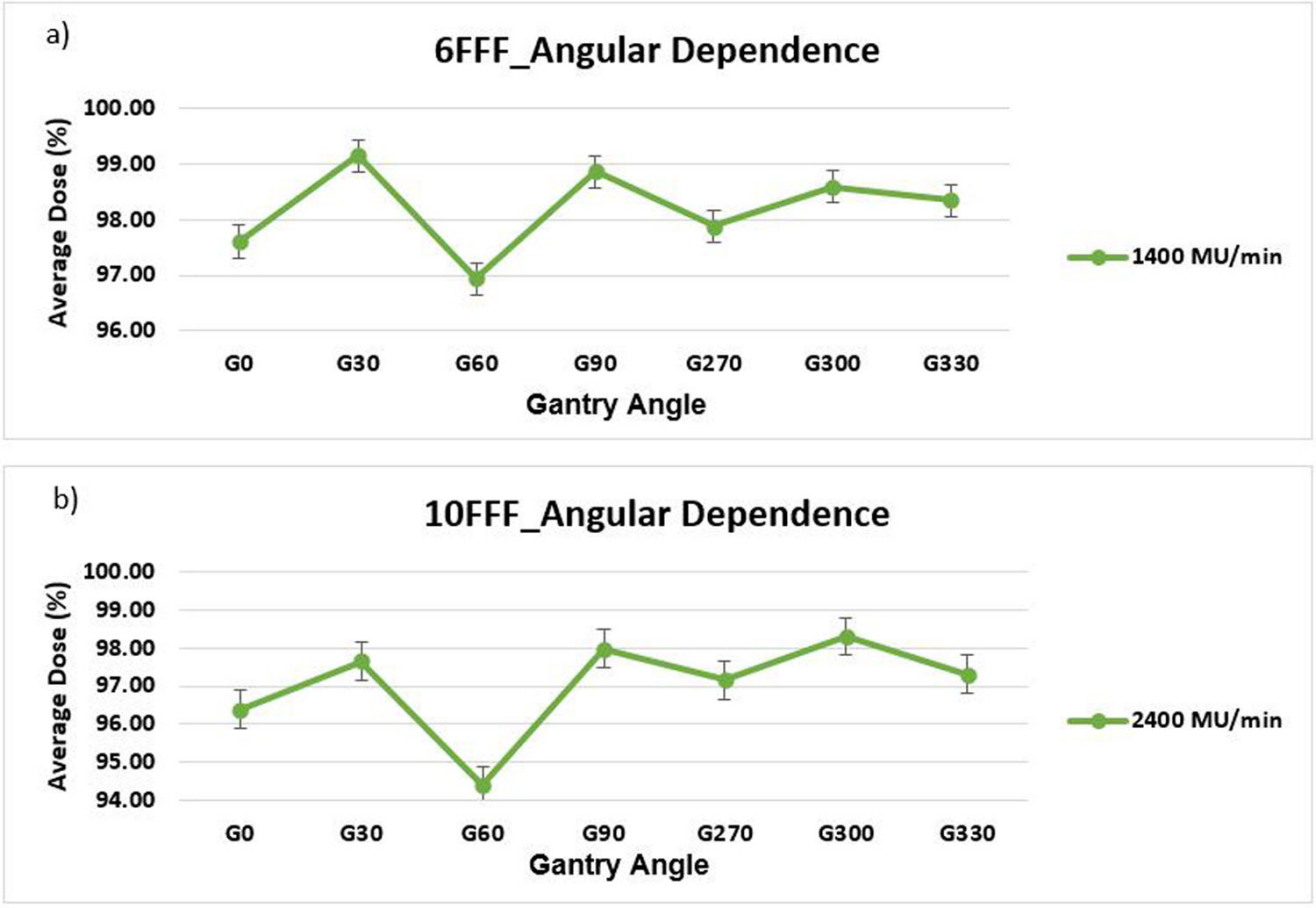
Angular dependence of ArcCheck phantom for (a) 6 MV FFF and (b) 10 MV FFF. The maximum deviation was 0.745 and 1.22 for 6 MV FFF and 10 MV FFF, respectively. G refers to gantry and number after G represents gantry angle

The angular response of ArcCHECK phantom was derived for 10 × 10 cm^2^ field size with 6 MV FFF and 10 MV FFF beams at 1400 MU/min and 2400 MU/min. The maximum deviation for 6 MV FFF was 0.745 and for 10 MV FFF was 1.22 (Figure 4).

### 3.2 Rotisserie test (symmetry of response with phantom rotated axially)

The results of Rotisserie test are presented in Table 1. The deviation is less than 1.5%.

**TABLE 1:** Results of the Rotisserie test

**Gantry angle**	**0**	**30**	**60**	**90**	**270**	**300**	**330**
Mean percentage	97.71	98.85	97.19	98.69	98.12	98.70	98.65
Standard deviation	1.26	0.68	1.44	1.07	1.41	0.82	0.79

Note: Mean percentage is the average of absolute dose of 6 diodes within 5 mm of the center of the irradiated field of the arccheck. 6 MV FFF and dose rate 1400 MU/min with10 × 10 cm^2^.

### 3.3 SD versus HD measurements

**FIGURE 5:**
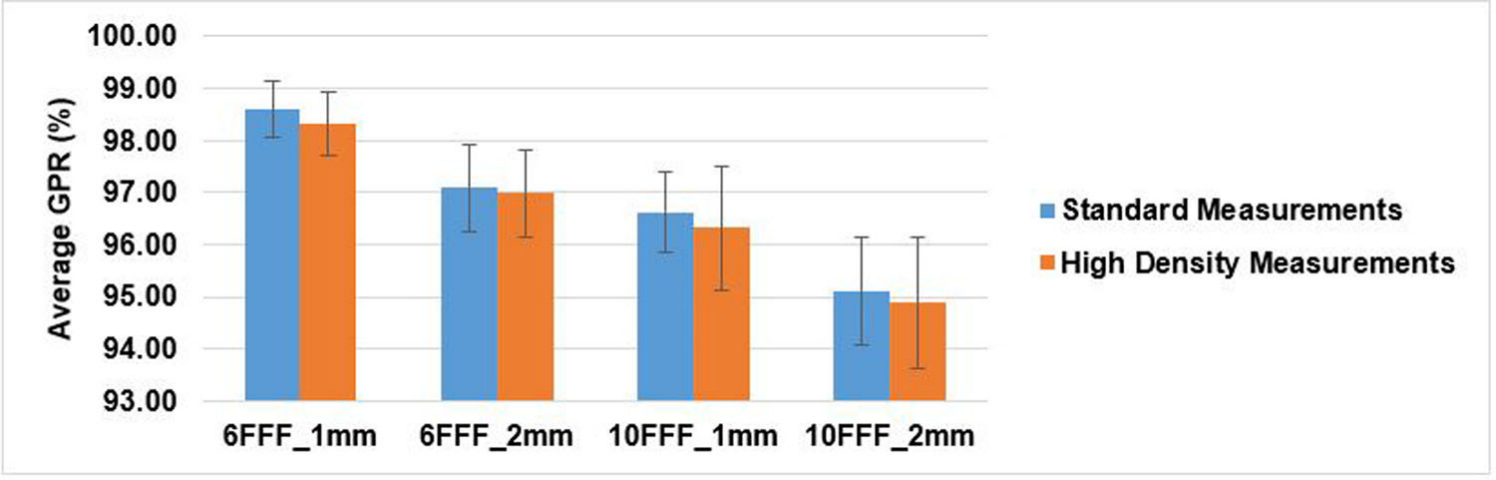
Note that 1 mm and 2 mm represent GS; 6 FFF and 10 FFF for 6 MV FFF and 10 MV FFF beam energies, respectively. As it is shown, SD measurements with 1 mm GS has better GPR for each energy

**FIGURE 6:**
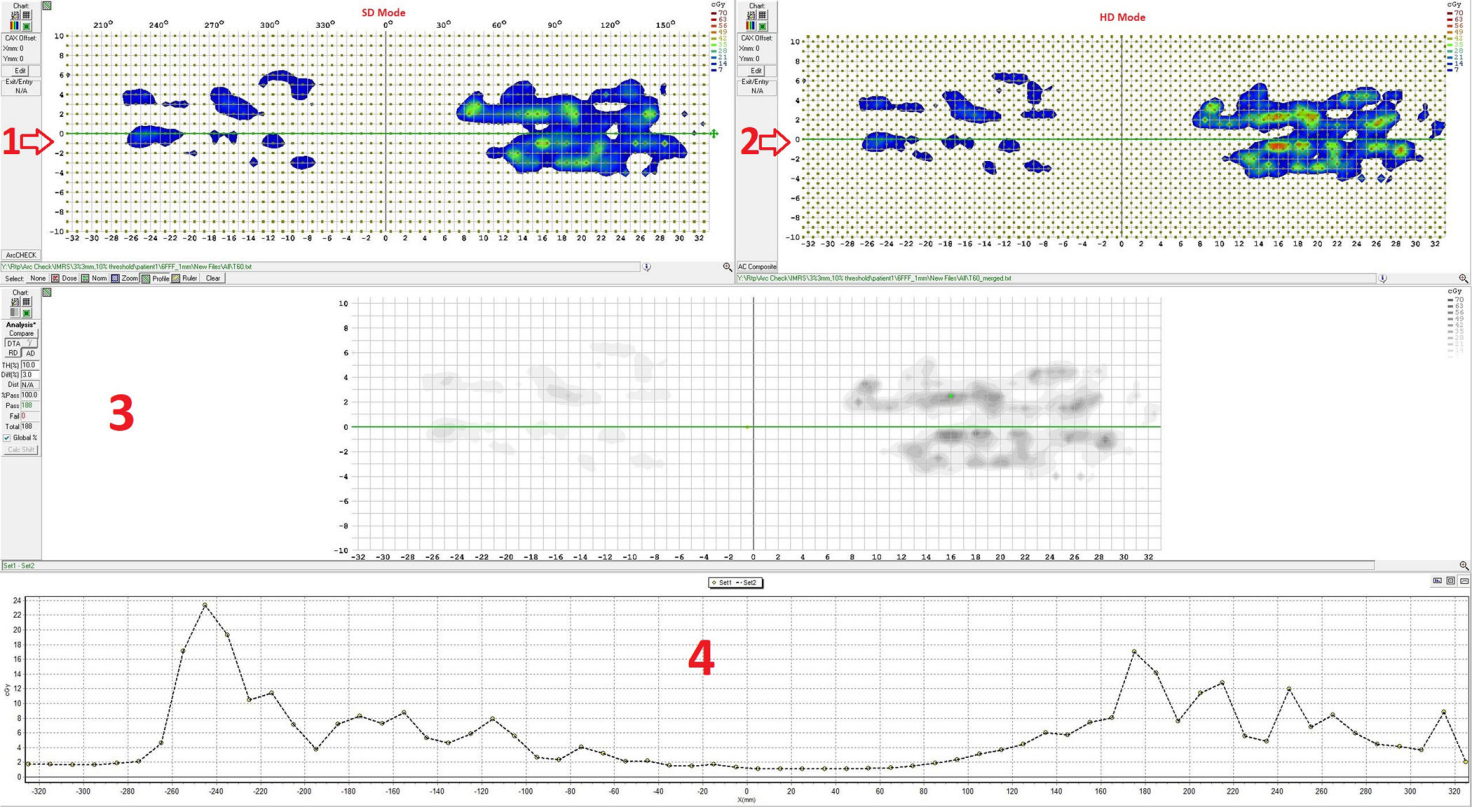
The visual representation of the intensity modulation within the treatment area for SD and HD modes. (1) set1 panel shows SD mode, (2) set2 panel displays HD mode, (3) compare panel: comparison between SD and HD mode and (4) profile and histogram panel which displays a profile across a selected axis. As we can see from part 3 and 4, SD and HD are perfectly matched with each other

As shown in Figures 5 and 6, using passing rate 95% with 3%/3 mm no noticeable difference in acceptance between HD and SD mode was found. One millimeter GS has higher GPR than 2 mm dose grid; the GPR of 6 MV FFF plans was higher than those of 10 MV FFF. Also, because gamma evaluation does a search around a detector. SD measurement with fine grid TPS computation provides better results because of less interpolation.

### 3.4 Second MU check and MF

**FIGURE 7:**
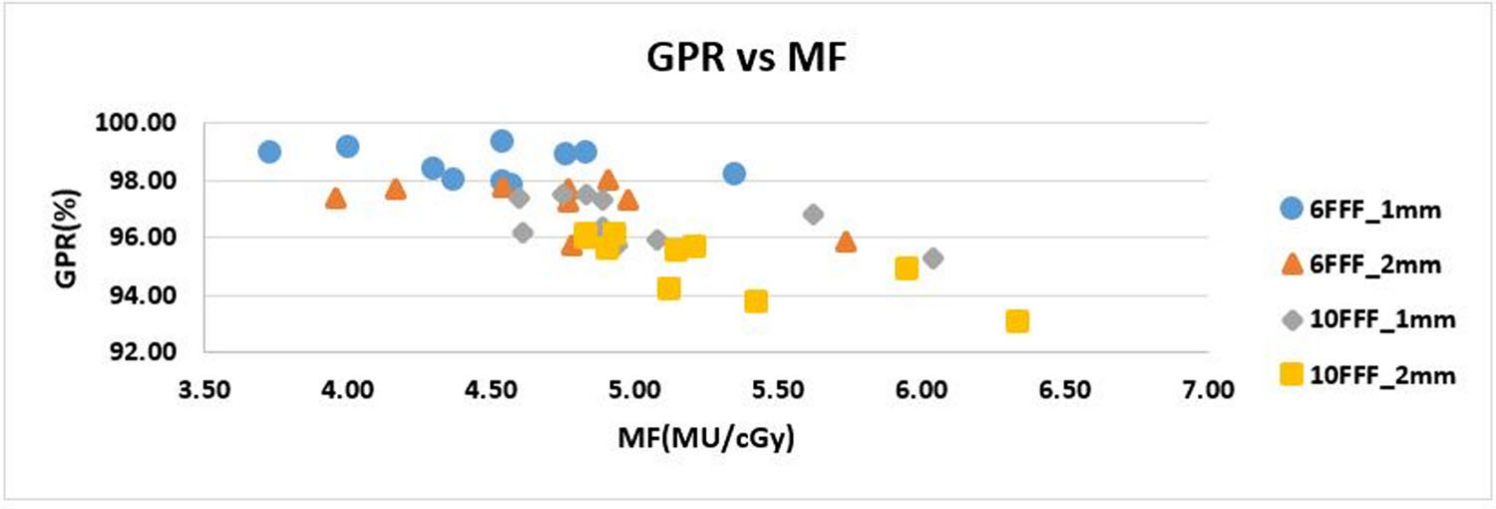
Note that 1 mm and 2 mm represent dose calculation GS; 6 FFF and 10 FFF for 6 MV FFF and 10 MV FFF beam energies, respectively. Negative correlation was observed between GPR and MF (−0.704 correlation coefficient)

The doses calculated with RadCalc agreed to within ±2% with the doses calculated by TPS. Modulation factor was calculated for each individual plan based on Equation (1). The range of MF for 6 MV FFF and 10 MV FFF with GS of 1 mm and 2 mm are presented in Table 2. Our results in Figure 7 show the correlation between GPR and MF: GPR (%) decreases with increasing total MF (correlation coefficient −0.704).

**TABLE 2:** MF of 6 FFF and 10 FFF with GS of 1 mm and 2 mm

	**Min MF**	**Max MF**	**Mean**	**STD**
6 FFF_1 mm	3.73	5.35	4.50	0.43
6 FFF_2 mm	3.96	5.74	4.75	0.46
10 FFF_1 mm	4.60	6.04	5.03	0.44
10 FFF_2 mm	4.83	6.34	5.27	0.48

Note: The maximum and minimum modulation factors were observed for 10 MV FFF with 2 mm GS and 6 MV FFF with 1 mm GS, respectively

### 3.5 Confidence interval

Error range of HD is narrower than SD mode due to higher density detector of HD than SD mode for measuring delivered dose (Figure 5); therefore, CI was calculated for SD measurements based on Equation (2). As shown in Table 3, GPR of HD mode is within the range of CI of that of SD for all energies and GS.

**TABLE 3:** Results of CI% calculation

	**Mean GPR% HD**	**Mean GPR% SD**	***N***	***N***	**CI%_SD**
6FFF 1 mm	98.31	98.60	202	372	98.60 ± 1.1
6FFF_2 mm	96.97	97.08	203	378	97.08 ± 1.58
10FFF 1 mm	96.31	96.62	192	362	96.62 ± 1.75
10FFF 2 mm	94.88	95.11	196	370	95.11 ± 2.07

*Note*: Note that 1 mm and 2 mm represent GS. 6 FFF and 10 FFF for 6 MV FFF and 10 MV FFF beam energies, respectively, where *n* is the number of data points used for data analysis with HD mode data acquisition and *n* is the number of sampled points for SD.

### 3.6 Confidence limit and detectability threshold

CL and DT calculated for each energy and dose grid based on Equations (3) and (4) respectively are presented in Table 4, demonstrating that DT of ArcCHECK decreases by increasing GS and energy. The DT value of the 6 MV FFF with dose grid of 1 mm was >95% for SD and HD modes, and DT value of 6 MV FFF with 1 mm and 2 mm GS was >95%. Overall, the VMAT QA pass rate of the present study was stable and of statistical significance for 6 MV FFF beams.

**TABLE 4:** Percentage of points passing gamma criteria of 3%/3 mm, averaged over the plans, with associated confidence limits

**(a)**	**Mean**	**Standard deviation**	**Maximum**	**Minimum**	**CL**	**DT**
6FFF_1 mm	98.60	1.154	100.000	95.5000	3.667	96.33
6FFF_2 mm	97.08	1.855	100.00	91.30	6.554	93.45
10FFF_1 mm	96.62	1.995	100.000	89.600	7.287	92.71
10FFF_2 mm	95.11	2.337	99.30	87.00	9.473	90.53

**(b)**	**Mean**	**Standard deviation**	**Maximum**	**Minimum**	**CL**	**DT**
6FFF_1 mm	98.31	1.324	100.00	93.30	4.285	95.72
6FFF_2 mm	97.04	0.834	97.90	95.33	4.595	95.41
10FFF_1 mm	96.31	2.203	99.30	86.60	8.004	92.00
10FFF_2 mm	94.88	1.248	96.27	92.26	7.566	92.43

*Note*: Standard measurements: (a) confidence limits for SD mode and (b) confidence limits for HD mode.

## 4 DISCUSSION

### 4.1 Dose‐rate, angular dependence, and Rotisserie test of ArcCHECK Phantom

There are many uncertainties in beam delivery verifications due to different categories of errors in treatment techniques such as random error, systematic error, and organ motion error, etc. Each of these has tolerance limits that impact the overall accuracy of the beam intensity delivery as planned.^16^ Therefore, each component involved in beam delivery must be evaluated to make sure they function within their tolerance limits. For this purpose, the Rotisserie test, angular and dose rate dependency tests were done on our ArcCHECK phantom. The dose rate data shown in Figure 3 demonstrate less than 1% dependence for 6 MV FFF and 10 MV FFF energies. The angular dependence of ArcCHECK data was less than 3% for 6 MV FFF, and less than 4% for 10 MV FFF as shown in Figure 4. The largest deviation was found at gantry angle of 60°. To determine if this deviation was Linac related anomaly or a feature of ArcCheck, we used an Exradin^®^ A16 ion chamber (Standard Imaging, Inc.) placed in the center of ArcCHECK phantom to conduct point dose measurement at different gantry angles including the 60° to check output constancy of our machine. The results showed robust response of the machine at different gantry angles. In a similar study, Saini and Zhu^17^ presented their work on the energy dependence of diode detectors for nominal energy ranging between Co‐60 and 17 MV. They concluded that the energy dependence does not vary with the diode type (*n*‐ or *p*‐type) but mainly is a function of the material of the buildup around the diodes and the geometry of the buildup material. Therefore, depending on the actual geometry of individual diode, its response may vary which indicates the importance of accuracy of angular dependence correction algorithm and array calibration.

### 4.2 Grid size effect on gamma index

Our data indicate GPR decreases by increasing GS from 1 to 2 mm. Similar observation was reported by Chun et al.^18^ and Tanooka et al.^19^ Chun et al showed that the average global GPR for VMAT with ArcCHECK decreased by increasing planning dose grid from 1 to 4 mm.

This difference is observed due to the inherent differences in the dose calculation for different GS in TPS and also the interpolation in SNC Patient program.^11^ Snyder et al.^20^ showed the effects of GS on dose calculation and DVH. They concluded that for small target, PTV coverage increases as GS decreases from 2.5 to 1 mm. As the GS is decreased below the size of the MLC width, modeling of the leaf tongue and groove effects may begin to appear in the calculated dose distribution. Above this point, each dose voxel will comprise an average of the entire leaf, and edge effects will likely play a smaller role as leaf transmission will dominate the dose component. As such, the accuracy of the modeling of the machine in the TPS may lead to more significant differences at the leaf edges. In RayStation, tongue and‐groove regions are only modeled for MLC leaf edges, where the edge is exposed into the MLC opening. For MLC leaf edges that are closed against another MLC leaf, no tongue‐and‐groove regions are added. This effect may enhance differences between measured and computed doses.^21^ In general, adequate MLC modeling considering leaf tip width, leaf tip end, transmission and tongue‐and‐groove is crucial in dose calculation in TPS, and several publications have showed the importance of accurate MLC modeling in IMRT dose calculations.^22^, ^23^

Moreover, when GS of TPS calculations is larger than 1 mm, the SNC Patient program automatically interpolates the calculated dose down to a 1 mm grid in order to reduce error in gamma calculation. The interpolation is recommended by TG‐218 to reduce the error in gamma calculation because the error is the function of the local dose gradient, the spacing between evaluated dose points, and the DTA criterion. When the dose spacing and DTA criteria are similar, the calculated error in gamma in areas of steep dose gradient is large.^24^ As a general rule, the spacing should be less than or equal to one‐third of Δ*d* (distance criteria in gamma calculation).^4^

### 4.3 Modulation factor

In 2018, Wu et al.^25^ presented compilation of dose verification in 924 IMRT plans which showed a negative correlation between total number of MU and GPR. The analysis of MF for each energy and GS are tabulated in Table 2. The correlation between GPR and MF was established to be −0.704, indicating that GPR decreases as total MU per plan increases, which agrees with Wu et al. As a result, GPR of 10 MV FFF is lower than 6 MV FFF due to higher MU. This may be attributed to the decreased off axis ratio of 10 MV FFF relative to 6 MV FFF which results in more monitor units required to generate the same uniform PTV coverage as that of 6 MV FFF. Also, our data indicate that in general MU of 2 mm GS is higher than 1 mm GS which result in lower GPR for 2 mm GS compared to 1 mm GS. The increased MU required for 2 mm GS may be attributed to the method of plan normalization chosen in this study, specifically normalizing to PTV *D*_95%_ at 100% Rx dose. Thus, the voxel volume averaging at the edge of the PTV, where the highest dose gradients are expected, results in increasing the number of monitor units required to achieve equivalent coverage. In the IMRS plans, where there are multiple small lesions using small fields to achieve sufficient coverage, the percentage of voxels at the “edge” of each target has a larger effect as the target size decreases. Subsequently, larger GSs caused a dose increase to the structures and required higher MUs to achieve the target coverage.^26^ Additional metrics for total plan modulation have been developed which include a number of dosimetric parameters such as MLC motion, gantry speed, changing dose rate, etc., which may warrant further study. Also, the evaluation of modulation factor presented in Equation (2) does not permit a per‐beam analysis of the GPR, which may provide more information for the determination of prediction of GPR.

### 4.4 SD versus HD mode

Data for both SD and HD mode have been compared with TPS calculated dose distributions using 95% passing rate with objectives of 3%/3 mm, indicating no significant differences between these modes of measurements (Table 1). However, a number of research publications showed how detector density can affect the GPR results.^27^, ^28^, ^29^ Common in these publications is that it was expected that GPR would be greater by increasing detector density. It is of note, however, that increasing detector density of ArcCHECK by a factor of 2 achieves a similar detector density to the MapCHECK2. The MapCheck2 is diode detector array with a resolution of 0.8 × 0.8 mm^2^, a diagonal detector spacing of 7.07 mm, and 1 cm parallel detector spacing. In 2017, Woon et al.^29^ observed a lack of correlation between GPR and percentage of dose error of the DVH metrics in MapCheck2 and ArcCHECK. Also, Montes et al.^30^ showed the GPR of SD and HD measurement modes of ArcCHECK phantom was almost the same and there were no differences in acceptance between both modes. Since no differences in acceptance between these modes for ArcCheck phantom were found and due to HD mode being more time consuming requiring two measurements and additional analysis steps, the SD mode is more clinically preferable.

### 4.5 Confidence interval

The CIs were calculated for both SD and HD datasets. The passing rate of HD mode falls within the range of CI of SD (Table 1). CI results indicate that SD measurements are good enough for performing patient‐specific QAs in the clinics.

### 4.6 Confidence limits

According to TG‐119, every institution should calculate the CL for their QA tests. It is expected that the CL calculated by the institution should be no greater than the CL recommended by TG‐119. Compared to the baseline provided by TG‐119, specifically the CL for diode array detectors should be no greater than 7%. The results of Table 4 show that only 6 MV FFF beams meet this goal. It may be noted however that the TG‐119 tests were performed with 6 MV flat beam, no data are available for non‐flat beams. It may be that removing the flattening filter from beam path, which affects parameters such as beam profile, head leakage, effective beam energy, dose rate, and beam steering and feedback, may result in recommending a larger CL under these measurement conditions. Therefore, further investigation is warranted.

## 5 CONCLUSION

We have analyzed several aspects of the pretreatment QA verification, required for successful clinical implementation of IMRS treatment of multiple brain metastases. While 1 mm GS provided more accurate dose distribution calculation in TPS, it also resulted in higher GPR due to less artifacts in gamma calculation. The observed negative correlation between GPR and the total number of MUs (directly translating to MF) indicates that for IMRT/VMAT planning, the total plan MU should be as low as possible to improve the GPR. Our results show that using the HD measurement mode of the ArcCHECK does not affect the GPR enough, at the tolerance levels set by our clinic, to result in any of the plans evaluated reaching the rejection threshold.  Thus, we conclude that HD mode has no clinical benefit justifying the increased workload. According to our results, for this treatment technique, 10 MV FFF with 2 mm GS results in the lowest GPR hence, 6 MV FFF with 1 mm GS is recommended for IMRS plans as it results in the highest GPR and presents other favorable dosimetric properties.

## CONFLICT OF INTEREST

The authors declare no conflict of interest.

## AUTHOR CONTRIBUTIONS

Elahheh Salari: Conceived and designed the analysis, collected the data, contributed data, performed the analysis, and wrote the paper. E. Ishmael Parsai: Conceived and designed the analysis, and wrote the paper. Diana Shvydka: Conceived and designed the analysis, and wrote the paper. Nicholas Niven: Conceived and designed the analysis, and wrote the paper.
